# Reduction of the Radiating Sound of a Submerged Finite Cylindrical Shell Structure by Active Vibration Control

**DOI:** 10.3390/s130202131

**Published:** 2013-02-06

**Authors:** Heung Soo Kim, Jung Woo Sohn, Juncheol Jeon, Seung-Bok Choi

**Affiliations:** 1 Department of Mechanical, Robotics and Energy Engineering, Dongguk University-Seoul, 30 Pildong-ro 1-gil, Jung-gu, 100-715 Seoul, Korea; E-Mail: heungsoo@dgu.edu; 2 Department of Mechanical Design Engineering, Kumoh National Institute of Technology, Daehak-ro 61, Gumi, 730-701 Gyeongbuk, Korea; E-Mail: jwsohn@kumoh.ac.kr; 3 Smart Structures and Systems Laboratory, Department of Mechanical Engineering, Inha University, 253 Young-Hyun Dong, Nam-Gu, 402-751 Incheon, Korea; E-Mail: daehanboy@gmail.com

**Keywords:** underwater cylindrical shell structure, macro fiber composite actuator, optimal controller, structural vibration control, structure-borne noise

## Abstract

In this work, active vibration control of an underwater cylindrical shell structure was investigated, to suppress structural vibration and structure-borne noise in water. Finite element modeling of the submerged cylindrical shell structure was developed, and experimentally evaluated. Modal reduction was conducted to obtain the reduced system equation for the active feedback control algorithm. Three Macro Fiber Composites (MFCs) were used as actuators and sensors. One MFC was used as an exciter. The optimum control algorithm was designed based on the reduced system equations. The active control performance was then evaluated using the lab scale underwater cylindrical shell structure. Structural vibration and structure-borne noise of the underwater cylindrical shell structure were reduced significantly by activating the optimal controller associated with the MFC actuators. The results provide that active vibration control of the underwater structure is a useful means to reduce structure-borne noise in water.

## 1. Introduction

In the last several decades, significant advances have been achieved in the field of smart materials and structures. One of the main applications of smart materials and structures is active vibration control to suppress undesirable structural vibration and noise. A smart structure has the capability to respond to changes in the external environment, as well as to a change of its internal environment. It incorporates smart materials that allow the change of system characteristics, such as stiffness or damping, in a controlled manner. Many types of smart materials are being developed as actuators and sensors, such as piezoelectric materials, shape memory alloys, electrorheological fluids, magnetorheological fluids, electrostrictive materials, magnetostrictive materials and electroactive polymers. In particular, piezoelectric materials are most commonly used as smart materials, owing to their quick response, wide bandwidth and easy implementation. Moreover, piezoelectric materials can be employed as both actuators and sensors, by taking advantage of direct and converse piezoelectric effects.

Crawley and de Luis provided pioneering work in this area, involving the development of the induced strain actuation mechanism [1]. Thereafter, numerous researches have been conducted to improve structural performance, based on the induced strain actuators and sensors [2]. However, most of these works were limited to plate type structures. Tzou *et al.* developed a distributed structural control scheme of elastic shells using spatially distributed modal piezoelectric actuators [3]. They formulated generic distributed feedback algorithms with spatial feedback functions. Lester and Lefebvre proposed piezoelectric actuator models for active sound and vibration control of cylinders [4]. Pan and Hansen reported the theoretical analysis of active control of harmonic power transmission in a semi-infinite cylinder, using a circumferential array of control forces, and a circumferential array of error sensors [5]. Maillard and Fuller presented analytical and experimental results of an investigation of active control of vibration and sound radiated from cylinders with piezoelectric actuators [6]. Kim *et al.* investigated active vibration control of a smart composite shell with surfaced bonded piezoelectric actuators [7]. Sohn *et al.* studied active vibration control of hull structures with linear quadratic Gaussian (LQG) theory [8]. Pan *et al.* reported a theoretical analysis of the active control of low-frequency radiated pressure from submarine hulls [9,10]. However, most of these works were limited to surface-bonded piezoelectric ceramic patches. Piezoelectric ceramic patches are very brittle, and not easy to use for curved geometry. To solve this problem, a Macro Fiber Composite (MFC) actuator, based on a sheet of rectangular piezoelectric ceramic fiber, was developed at the NASA Langley Research Center [11,12]. The MFC actuator is flexible, and therefore applicable to curved structures. In-plane poling with d33 property can be achieved by an interdigitated electrode, which produces more induced actuating strain than possible with a monolithic piezoelectric ceramic patch. Azzouz *et al.* investigated finite element modeling of an MFC actuator [13], and Sodano *et al.* studied applications of MFC actuators in structural vibration control [14]. Choi *et al.* presented active vibration control of pre-twisted rotating composite thin-walled beam with MFC actuators [15]. They used a negative velocity feedback control algorithm to suppress a pre-twisted rotating blade. Dano and Julliere reported the use of MFC actuators to control thermally induced deformations in laminated composite structures [16]. Barkanov *et al.* investigated the active twist control of a helicopter rotor blade using an MFC actuator to reduce vibration and noise, without any complex mechanism in the rotating systems [17]. Binette *et al.* studied the shape control of composite structures using MFC actuators [18]. They used MFC actuators to compensate for thermally-induced distortion of a sandwich plate subjected to a through-the-thickness thermal gradient. Vadiraja and Sahasrabudhe proposed the structural modeling of a rotating pre-twisted composite beam with embedded MFC actuators and sensors, using higher shear deformation theory [19,20]. They used a LQG control algorithm to reduce the structural vibrations of the box beam. Bilgen *et al.* demonstrated a variable camber airfoil using MFC actuators [21,22]. Sohn *et al.* reported active vibration control of a smart hull structure using MFC actuators [23,24]. All of these works involve active vibration/noise control of smart structures using MFC actuators in air conditions. Zhang *et al.* investigated underwater sound radiation control of a stiffened plate structure by the active vibration isolation technique [25], and evaluated their scheme experimentally [26]. Caresta reported the active control of sound radiation of a submarine hull structure in theoretical bending vibration [27]. Experimental research on the active vibration control of underwater structure is rare.

Consequently, the main purpose of this work is to actively control the imposed vibration of an underwater cylindrical shell structure using MFC actuators, and to experimentally investigate the reduction of structure-borne noise due to the vibration control effect. Finite element modeling was developed to obtain a state space equation for the active control algorithm. The optimal control algorithm was designed and experimentally implemented to suppress structural vibration in water. It has been demonstrated that the imposed vibration of the underwater cylindrical shell structure was suppressed significantly, based on the designed optimal control algorithm. In addition, it has been measured that the structure-borne noise was effectively reduced, by actively controlling the vibration using MFC actuators.

## 2. Dynamic Modeling of Underwater Cylindrical Shell Structure

**Figure 1.**
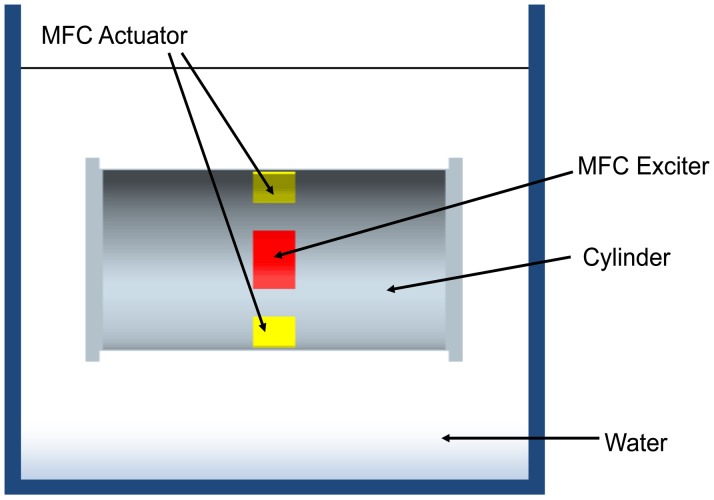
Schematic diagram of the proposed underwater cylindrical shell structure.

A schematic diagram of the proposed underwater cylindrical shell structure for vibration control is shown in Figure 1.

A simple end-capped cylindrical shell structure is considered as the host structure, which can be considered as the simple model of a submarine. MFC actuators are bonded on the surface of the host structure, and perfect bonding is assumed between the host structure and actuators. The structure is considered in the water, with free-free boundary conditions.

Finite element modeling of the underwater cylindrical shell structure is conducted first. The underwater cylindrical shell structure is divided into three parts, namely the cylinder, MFC actuators and water. The cylinder and MFC actuators are finite, but the fluid is infinite in dimension. In mathematical modeling of the underwater cylindrical shell structure, the fluid-structure interaction must be considered. The equations of motion of the underwater cylindrical shell structure can be expressed as follows [28]:

(1) $${\text{M}}_{S}\ddot{\text{U}}+{\text{C}}_{S}\dot{\text{U}}+{\text{K}}_{S}\text{U}={\text{F}}_{S}+{\text{F}}_{I}$$

where **U** is the nodal displacement. The matrices **M***_S_*, **C***_S_* and **K***_S_* are mass, damping and stiffness matrices of the host structure, respectively. The damping matrix **C***_S_* is assumed to be proportional to the mass and the stiffness matrices. The force **F***_S_* is an external mechanical force applied to the host structure. The force **F***_I_* is an external fluid force generated by the fluid-structure interaction. The analysis of fluid-structure interaction is a procedure to find **F***_I_* as a function of structural response. If the cylindrical shell structure under water was vibrating in low frequency bandwidth, the surrounding fluid can be assumed as an ideal fluid. Then, the tangential shear force occurring on the surface of the fluid-structure interface can be neglected. Therefore, there is only the normal force generated on the surface of the fluid-structure interface, *Γ_S_*. Finally, **F***_I_* is expressed as follows:

(2) $${\text{F}}_{I}=\int_{\Gamma_{S}}N^{T}\bar{n}\text{P}dS$$

where *N* is the shape function to approximate the pressure at the given element. **P** is the pressure vector obtained from the fluid-structure interaction. *n̅* is a unit normal vector on the surface of the fluid-structure interface, and *dS* is an infinitesimal area of the fluid-structure interface. The pressure, **P**_,_ is solved by applying the boundary element method, based on the inverse formulation of Euler's solution. Then, one can obtain:

(3) $${\text{F}}_{I}=-{\text{M}}_{F}\ddot{\text{U}}$$

where **M***_F_* is an added mass matrix. Substituting Equation (3) into Equation (1), the equation of motion for the fluid-structure interaction is expressed as follows:

(4) $$({\text{M}}_{S}+{\text{M}}_{F})\ddot{\text{U}}+{\text{C}}_{S}\dot{\text{U}}+{\text{K}}_{S}\text{U}={\text{F}}_{S}$$

Now electro-mechanical coupling of the full structure and MFC actuators is considered. After the application of the variational principle and finite element discretization, the coupled finite element equations of motion can be expressed as follows:

(5) $$\left[\begin{array}{cc} {\text{M}}_{S}+{\text{M}}_{F} & 0\\ 0 & 0 \end{array}\right]\left\{\begin{array}{c} \ddot{\text{U}}\\ \ddot{\phi } \end{array}\right\}+\left[\begin{array}{cc} {\text{C}}_{S} & 0\\ 0 & 0 \end{array}\right]\left\{\begin{array}{c} \text{U}\\ \dot{\phi } \end{array}\right\}+\left[\begin{array}{cc} {\text{K}}_{uu} & {\text{K}}_{u\phi }\\ {\text{K}}_{\phi u} & {\text{K}}_{\phi \phi } \end{array}\right]\left\{\begin{array}{c} \text{U}\\ \phi \end{array}\right\}=\left\{\begin{array}{c} {\text{F}}_{S}\\ {\text{F}}_{\phi } \end{array}\right\}$$

where *ϕ* is the electric potential vector. The matrices **K***_uϕ_* and **K***_ϕu_* are stiffness matrices due to piezoelectric-mechanical coupling (converse and direct piezoelectric effects). Their presence allows piezoelectric materials to produce mechanical actuation forces under input voltages, or electric signals under mechanical deformations. The matrix **K***_ϕϕ_* is the stiffness matrix resulting from the electric field. The stiffness coupling effects can influence the equilibrium position if a steady state exists. The vectors **F***_S_* and **F***_ϕ_* are force vectors due to the mechanical and electric fields, respectively. After static condensation, the equations of motion can be reduced, and can be expressed in terms of nodal displacement only:

(6) $$\text{M}\ddot{\text{U}}+\text{C}\dot{\text{U}}+\text{KU}=\text{F}$$

where:

(7) $$\text{M}={\text{M}}_{S}+{\text{M}}_{F},\text{K}={\text{K}}_{uu}-{\text{K}}_{u\phi }{{\text{K}}_{\phi \phi }}^{-1}{\text{K}}_{\phi u},\text{F}={\text{F}}_{S}-{\text{K}}_{u\phi }{{\text{K}}_{\phi \phi }}^{-1}{\text{F}}_{\phi }$$

The above reduced equations of motion are coupled with each other. The mesh size must be fine to obtain accurate dynamic responses of the underwater cylindrical shell structure. However, the feedback control algorithm requires a small size of the system matrix, due to the limitation of computer performance. The size of the above system must be reduced for the active feedback control. The most commonly used method to reduce the size of the system is modal reduction. The above coupled equations of motion are first solved for un-damped free vibration. The mode shapes are extracted, and assembled as a modal matrix Φ. Then, the modal matrix is used to transform the global displacement vector **U** to the modal displacement vector *η*, as follows:

(8) U=Φη

Substituting Equation (8) into Equation (6) with modal reduction, the decoupled dynamic equation of motion for the feedback control system is obtained:

(9) $$\hat{\text{M}}\ddot{\eta }+\hat{\text{C}}\dot{\eta }+\hat{\text{K}}\eta =\hat{\text{F}}$$

where:

(10) $$\hat{\text{M}}=\Phi^{T}({\text{M}}_{S}+{\text{M}}_{F})\Phi ,\hat{\text{C}}=\Phi^{T}\text{C}\Phi ,\hat{\text{K}}=\Phi^{T}\text{K}\Phi ,\hat{\text{F}}=\Phi^{T}\text{F}$$

The matrices **M̂**, **Ĉ** and **K̂** are modal mass, modal damping and modal stiffness matrices, respectively. After the modal reduction, the state space equation is derived for the design of the active feedback control algorithm as follows:

(11) $$\dot{\text{x}}=\text{Ax}+\text{B}{\text{u}}_{c}$$

where:

(12) $$\text{A}=\left[\begin{array}{cc} 0 & \text{I}\\ -{\hat{\text{M}}}^{-1}\hat{\text{K}} & -{\hat{\text{M}}}^{-1}\hat{\text{C}} \end{array}\right],\text{B}=\left[\begin{array}{c} 0\\ {\hat{\text{M}}}^{-1}\hat{\text{F}} \end{array}\right],\text{x}={\left[\eta \;\dot{\eta }\right]}^{T}$$

In the feedback control algorithm, **x** is the control state vector, **A** the control system matrix, **B** the control input matrix, and **u***_c_* the control input of the system. If we consider fundamental *n* modes for the modal reduction, the system matrix, control input matrix and state vector are expressed as follows:

(13) $$\begin{array}{l} \text{A}=\left[\begin{array}{ccccccc} 0 & 1 & & & & & \\ -\omega_{1}^{2} & -2\zeta_{1}\omega_{1} & & & & & \\ & & 0 & 1 & & & \\ & & -\omega_{2}^{2} & -2\zeta_{2}\omega_{2} & & & \\ & & & & \ddots & & \\ & & & & & 0 & 1\\ & & & & & -\omega_{n}^{2} & -2\zeta_{n}\omega_{n} \end{array}\right],\text{B}=\left[\begin{array}{c} 0\\ {\hat{f}}_{1}\\ 0\\ {\hat{f}}_{2}\\ \vdots \\ 0\\ {\hat{f}}_{n} \end{array}\right]\\ \text{x}={\left[\begin{array}{ccccccc} \eta_{1} & {\dot{\eta }}_{1} & \eta_{2} & {\dot{\eta }}_{2} & \cdots & \eta_{n} & {\dot{\eta }}_{n} \end{array}\right]}^{T} \end{array}$$

where *ω_i_* and *ζ_i_* are the *i-th* natural frequency and damping ratio of the underwater cylindrical shell structure, and *f̂_i_* is an actuating force vector at the *i-th* mode.

## 3. Design of the Optimal Controller

An optimal control algorithm is designed for active vibration control of the underwater cylindrical shell structure. The sensor noises and system disturbances are also considered for the actual implementation of the cylindrical shell structure. The control purpose is to regulate the unwanted vibrations of the cylindrical shell structure. Thus, the performance index to be minimized is chosen as follows:

(14) $$J=\int_{0}^{\infty }\left\{\text{x}{\left(t\right)}^{T}\text{Qx}(t\text{)}+\text{u}{\left(t\right)}^{T}\text{Ru}(t)\right\}dt$$

In the above equation, **Q** is the state weighting semi-positive matrix, and **R** is the input weighting positive matrix. Since the system(**A,B**) in Equation (11) is controllable, one can obtain the following linear quadratic regulator (LQR):

(15) $$\text{u}(t)=-{\text{K}}_{G}\eta (t)$$

Here, **K***_G_* is the state feedback gain matrix, and can be obtained from the following equation:

(16) $${\text{K}}_{G}=(\text{R}+{\text{B}}^{T}\text{PB}){\text{B}}^{T}\text{PA}$$

where **P** is the solution of the following algebraic Riccati equation:

(17) $${\text{A}}^{T}\text{PA}-\text{P}-{\text{A}}^{T}\text{PB}{\left(\text{R}+{\text{B}}^{T}\text{PB}\right)}^{-1}{\text{B}}^{T}\text{PA}+\text{Q}=0$$

Since the states *η_i_*(*t*) and *η̇_i_*(*t*) of LQR are not available from direct measurement of the current underwater cylindrical shell structure, a Kalman-Bucy Filter (KBF) is formulated. The KBF is a state estimator, which is considered optimal in the statistical sense. The state space model, considering observation modes, can be given by:

(18) $$\begin{array}{l} \dot{\text{x}}(t)=\text{Ax}(t)+\text{Bu}(t)+{\text{w}}_{1}\\ \text{y}(t)={\text{C}}_{y}\text{x}(t)+{\text{w}}_{2} \end{array}$$

where **w**_1_ and **w**_2_ are uncorrelated white noise characterized by covariance matrices **V**_1_ and **V**_2_, as follows:

(19) $$\begin{array}{l} \text{Cov}({\text{w}}_{1},{\text{w}}_{1}^{T})={\text{V}}_{1}\\ \text{Cov}({\text{w}}_{2},{\text{w}}_{2}^{T})={\text{V}}_{2}\\ \text{Cov}({\text{w}}_{1},{\text{w}}_{2}^{T})=0 \end{array}$$

The estimated state, **x̂**(*t*), can be obtained as follows:

(20) $$\dot{\hat{\text{x}}}(t)=\text{A}\hat{\text{x}}(t)+\text{Bu}(t)+\text{L}(\text{y}(t)-{\text{C}}_{y}\hat{\text{x}}(t))$$

where:

(21) $$\text{L}=\text{A}\Sigma {\text{C}}^{T}{\left(\text{C}\Sigma C^{T}+{\text{V}}_{2}\right)}^{-1}$$

In the above equation, **L** is the observer gain matrix, and ∑ is the solution of the following observer Riccati equation:

(22) $$\text{A}\Sigma {\text{A}}^{T}-\Sigma -\text{A}\Sigma {\text{C}}^{T}{\left(\text{C}\Sigma {\text{C}}^{T}+{\text{V}}_{2}\right)}^{-1}\text{C}\Sigma {\text{C}}^{T}+{\text{V}}_{1}=0$$

Using the estimated states, the control input is obtained as follows:

(23) $$\text{u}(t)=-{\text{K}}_{G}\hat{\text{x}}(t)$$

**Figure 2.**
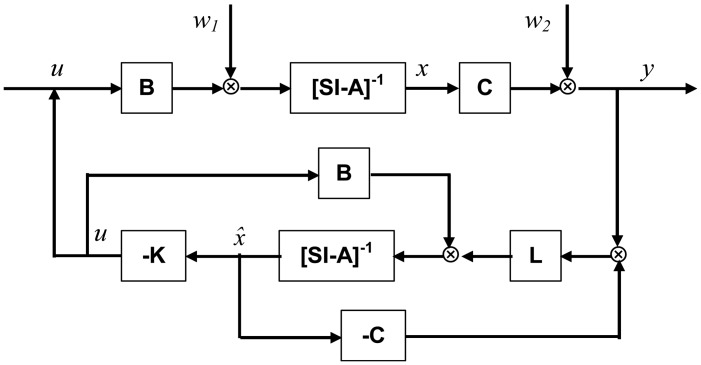
Block diagram of LQG control algorithm.

A block diagram of the proposed LQG controller is presented in Figure 2.

## 4. Evaluation of Control Performance

**Figure 3.**
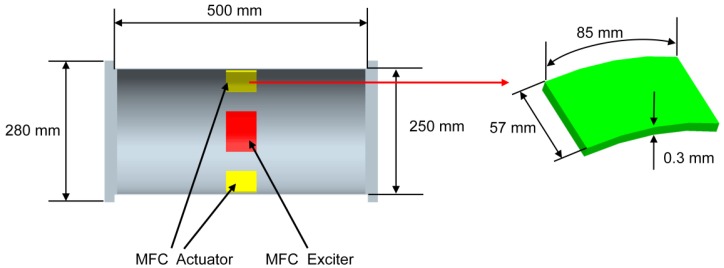
Geometry of the end-capped cylindrical shell structure with surface bonded MFC actuators.

This study investigates active vibration control of the underwater cylindrical shell structure. The geometries of the proposed cylindrical shell structure and MFC actuators are given in Figure 3. The length, radius and thickness of the host structure are 500 mm, 125 mm and 2 mm, respectively. The radius and thickness of the end-cap are 140 mm and 10 mm, respectively. The length, width and thickness of the MFC actuator are 85 mm, 57 mm and 0.3 mm, respectively. The thickness of the bonding layer is neglected. Aluminum is used for the cylinder, and the material properties of the aluminum, MFC and water are listed in Table 1.

### 4.1. Modal Characteristics of the Underwater Cylindrical Shell Structure

**Figure 4.**
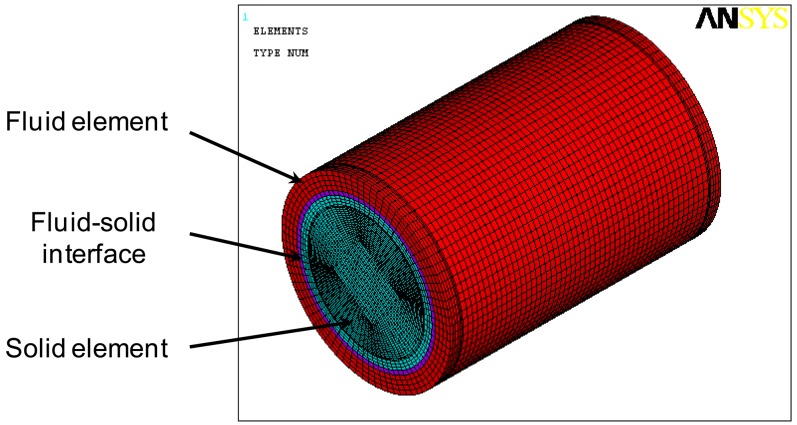
Finite element mesh configuration.

Modal characteristics of the proposed underwater cylindrical shell structure were first investigated by using the commercial finite element analysis package ANSYS. The finite element mesh configuration is presented in Figure 4. An eight-node structural solid brick element (SOLID45) is used for the cylindrical shell structure, an eight-node coupled field solid brick element (SOLID5) for the MFC actuator, an eight-node fluid brick element (FLUID30) for the water, and an eight-node coupled field fluid brick element (FLUID80) for the fluid-structure interface, respectively.

**Figure 5.**
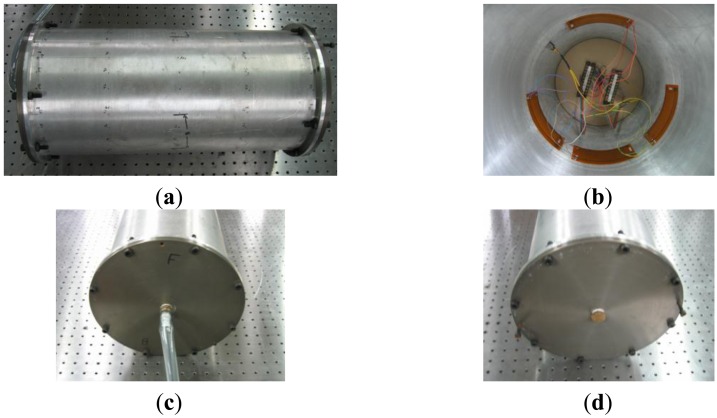
Photographs of the manufactured cylindrical shell structure. (**a**) Outside view, (**b**) Inside view, (**c**) Front view, and (**d**) Rear View.

**Figure 6.**
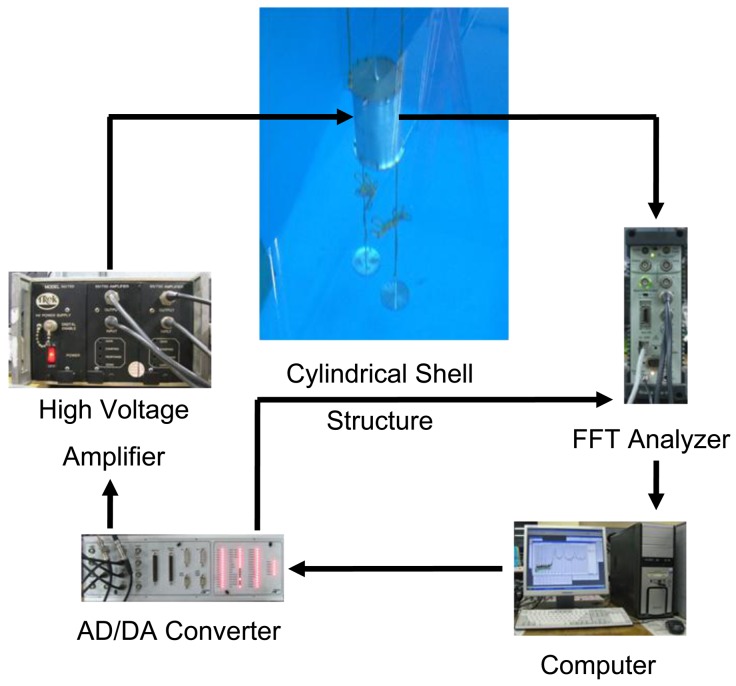
Experimental apparatus for the modal test of the underwater cylindrical shell structure.

An experimental test was also conducted to validate the finite element modeling. The manufactured end-capped aluminum cylindrical shell structure is shown in Figure 5. Each end-cap was attached to the host cylinder by bolt. A hole for electric wires was made at the center of one end-cap. The experimental apparatus for the modal test is shown in Figure 6. In order to submerge the cylindrical shell structure in the water tank, two weights were installed, and connected to the cylindrical shell structure by ropes, as shown in the figure. The top of the cylindrical shell structure was submerged 1 m below the surface of the water. An FFT analyzer (PULSE 3560B, Brüel & Kjær), accelerometer, MFC actuators and MFC sensors were used for the modal test. The dynamic characteristics of the underwater aluminum cylindrical shell structure with MFC actuators were measured. Three MFCs were used as actuators and sensors. One MFC was used as an exciter. Three MFCs were attached to the inside surface of the cylindrical shell structure at the center of the longitudinal direction, with equal spacing (120 degrees) about the circumferential direction, as shown in Figure 5(b). The exciting MFC was attached between the 1st and 2nd MFCs. The underwater cylindrical shell was excited by sine sweeping up to 700 Hz, and structural vibration was simultaneously measured by the three MFCs and accelerometer.

**Figure 7.**
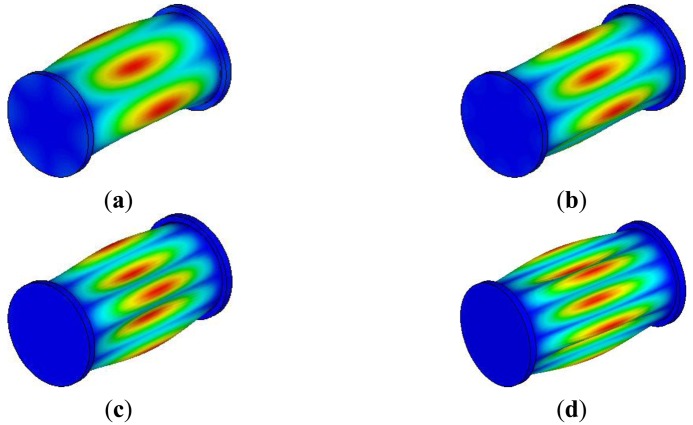
Fundamental mode shapes of underwater cylindrical shell structure. (**a**) 1st mode, (**b**) 2nd mode, (**c**) 3rd mode, and (**d**) 4th mode.

The fundamental mode shapes of the underwater cylindrical shell structure are presented in Figure 7. The mode shapes were not changed, compared to those of cylindrical shell structures in the air. However, natural frequencies of the cylindrical shell structure under water decreased a lot, compared to those of the cylindrical shell structure in the air. The comparisons of natural frequencies of the cylindrical shell structure in air and in water are presented in Table 2. Natural frequencies were obtained by finite element analysis. The decreasing rates of fundamental natural frequencies range from 44 to 67 percent. This is because of an added fluid mass in Equation (4).

**Figure 8.**
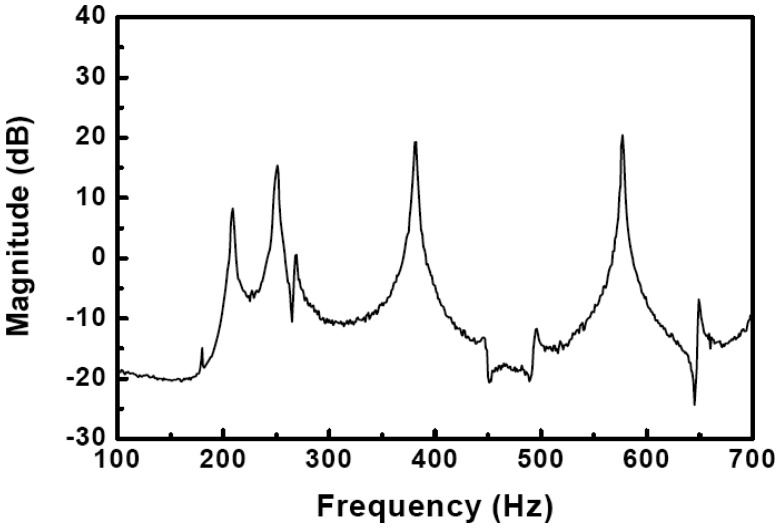
Frequency response of the underwater cylindrical shell structure.

Modal analysis of the finite element modeling is evaluated by the modal test. The experimental frequency response of the underwater cylindrical shell structure is presented in Figure 8. The fundamental natural frequencies obtained by FEA and experiment are presented in Table 3. The experimental natural frequencies are about 10 percent larger than the numerical ones. This might be caused by the rope boundary conditions in water, and the bolting of end-caps, which were not considered in the finite element modeling. Although the experimental natural frequencies are a little bit larger than the numerical ones, the FEA model accurately predicts the dynamic characteristics of the underwater cylindrical shell structure. Therefore, it is concluded that the FEA model can be used as a system matrix in the active vibration control. Since higher frequencies of structural responses are easily decayed by the structural damping, six fundamental natural frequencies and mode shapes are used to construct the system matrix for the active vibration control.

### 4.2. Active Vibration Control

**Figure 9.**
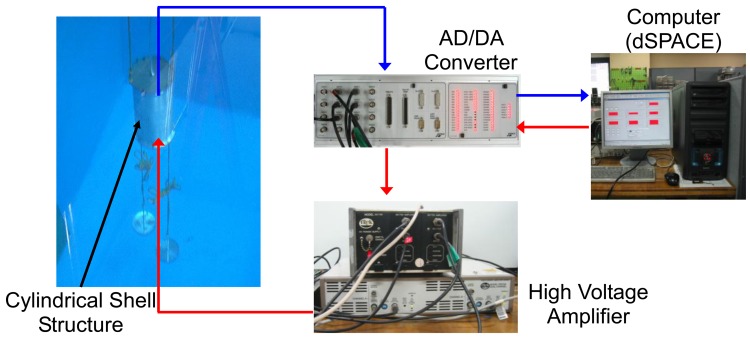
Experimental setup for active vibration control.

Now, active vibration control performance of the underwater cylindrical shell structure is studied. The experimental apparatus for the active vibration control is presented in Figure 9.

**Figure 10.**
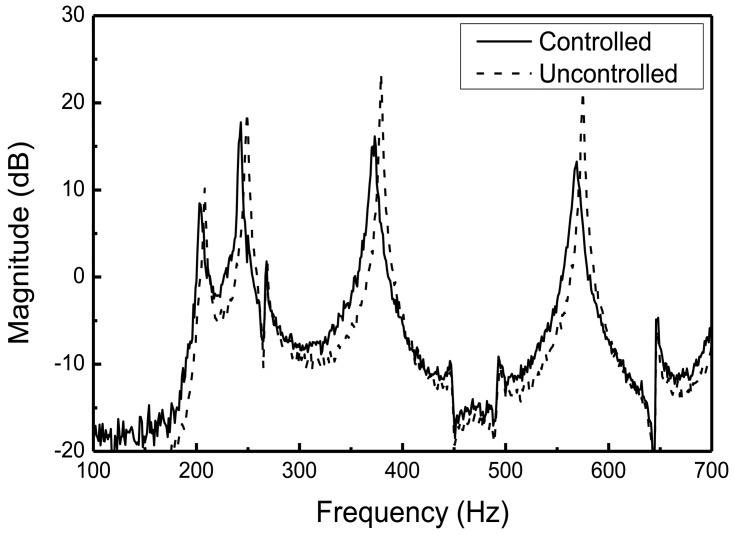
Frequency responses of the underwater cylindrical shell structure with and without active control.

The excitation signal, which is generated from a personal computer, was sent to the MFC exciter through a high voltage amplifier. Structural vibration was measured by the collocated MFC sensors, and proper control inputs were determined based on the designed LQG control algorithm in the dSPACE control system. The weighting matrices in Equation (14) were given as *diag*(**Q**) = 3 × 10^7^ and *diag*(**R**) = 1, respectively. The proportional damping ratio was assumed as 0.2 percent for each mode. After applying random excitation to the structure, the frequency responses of the cylindrical shell structure with and without active controller are presented in Figure 10.

**Figure 11.**
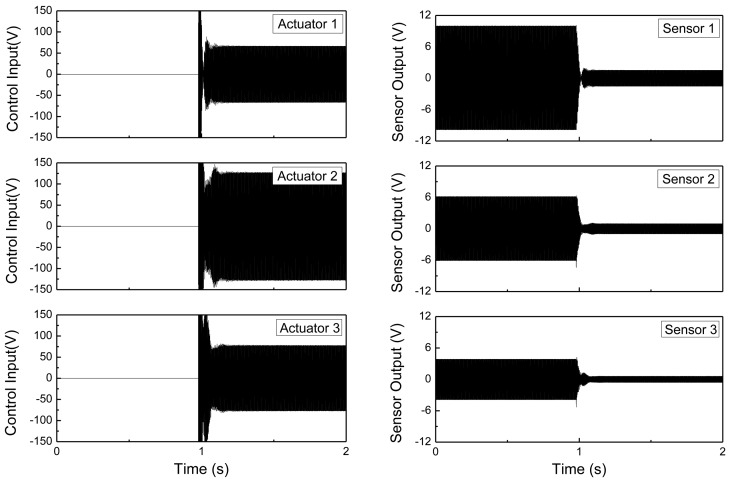
Actuator input voltages and control response under 3rd mode excitation.

The maximum vibration reduction was 8 dB at the fourth resonant frequency, and the minimum reduction was 1.5 dB at the second resonant frequency. The vibration reductions at the 1st and 2nd resonant frequencies were smaller than those of the 3rd and 4th resonant frequencies. This is because of the locations of MFC actuators. The 3rd and 4th modes of the cylindrical shell structure can develop large strains of MFC actuators, whereas the 1st and 2nd modes do not. This reveals that optimum placements of the MFC actuators are important to improve the performance of the active vibration control of the underwater cylindrical shell structure. Vibration control performances under 3rd mode excitation are presented in the time domain in Figure 11. By applying a control input with a maximum limit of 150 V, the structural vibration can be effectively suppressed for the underwater cylindrical shell structure.

**Figure 12.**
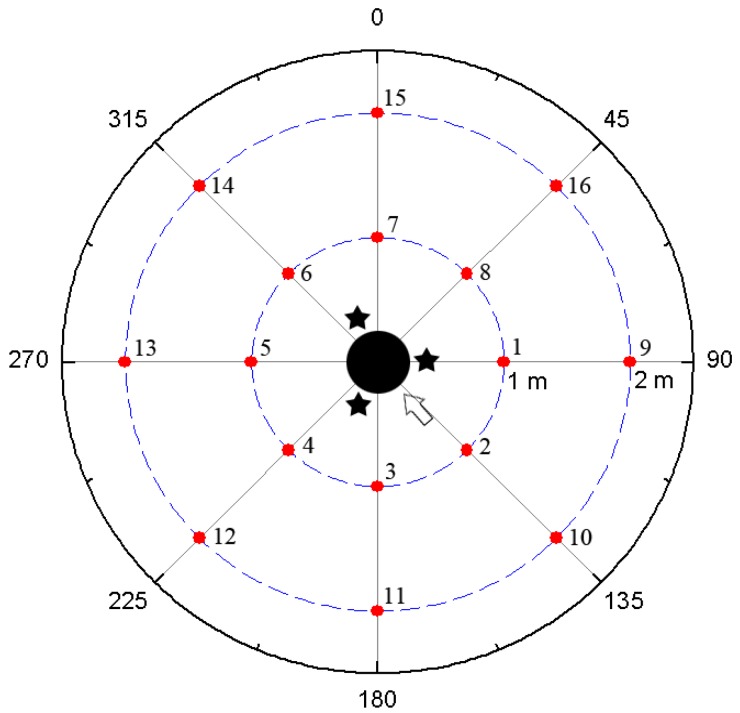
Positions of hydrophones.

Structure-borne noise due to active vibration control was also measured by hydrophone. The cylindrical shell structure was located at the center of the water pool, and sixteen hydrophones were radially located, as shown in Figure 12.

**Figure 13.**
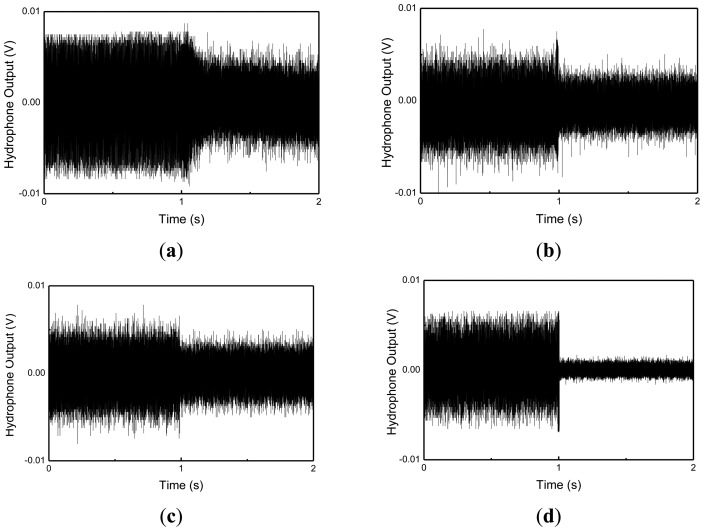
Measured radiating sound under 3rd mode excitation. (**a**) Hydrophone 2, (**b**) Hydrophone 6, (**c**) Hydrophone 10, and (**d**) Hydrophone 14.

**Figure 14.**
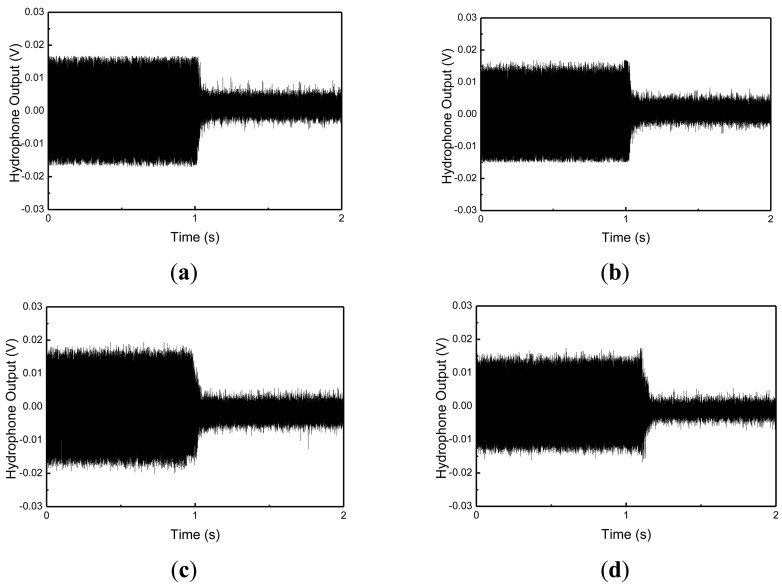
Measured radiating sound under 4th mode excitation (**a**) Hydrophone 2, (**b**) Hydrophone 6, (**c**) Hydrophone 10, and (**d**) Hydrophone 14.

The hydrophone was located with equal spacing (45 degree) about the circumferential direction, and at 1 m and 2 m distances from the cylindrical shell structure, respectively. Stars represent the positions of the MFC actuators, and an arrow represents the position of the MFC exciter in the figure. The radiating sounds measured at hydrophones 2, 6, 10 and 14 under 3rd and 4th mode excitation are presented in Figures 13 and 14.

When a hydrophone is close to the exciting MFC, the radiating sound is larger than that at other positions. For 3rd mode excitation, as shown in Figure 13, the radiated sounds at hydrophone 6 and 14 were suppressed 5% and 30% more than those of at hydrophone 2 and 10, respectively. For 4th mode excitation in Figure 14, the radiated sounds at hydrophone 6 and 14 were suppressed 3% and 5% more than those of at hydrophone 2 and 10, respectively. The results represent that, if the hydrophone was close to the MFC actuators, the radiating sounds were more suppressed. The results presented in this work provide that active vibration control of underwater cylindrical shell structure can effectively reduce structure-borne noise.

## 5. Conclusions

Active vibration control of an underwater cylindrical shell structure was investigated in this paper. Finite element modeling of the underwater cylindrical shell structure was developed, and dynamic characteristics were obtained by using the commercial finite element package, ANSYS. The fluid-structure interaction was modeled as an added fluid mass. Modal reduction of the given underwater cylindrical shell structure was conducted to obtain the reduced state space equations, for the design of an active feedback control algorithm. A LQG controller was designed, based on the reduced system matrix. Three MFCs were used as actuators and sensors, and one MFC was used as an exciter. Vibration control of the proposed system was evaluated by lab scale experiments. Structural vibration of the underwater cylindrical shell structure was suppressed by the designed optimal control algorithm associated with the MFC actuators. Structure-borne noise was also reduced by the active vibration control. It is concluded that active vibration control of the underwater cylindrical shell structure is useful for the reduction of structural vibration, and of structure-borne noise as well.

## Figures and Tables

**Table 1.** Material properties of the aluminum, MFC and water.

**Aluminum**			
Young's modulus (*E*)	68 [GPa]	Density (*ρ*)	2,698 [kg/m^3^]
Poisson ratio (*ν*)	0.32		
**MFC (poling direction: 1)**			
Young's modulus 1 direction (*E*_1_)	30.34 [GPa]	Young's modulus 2 direction (*E*_2_)	15.86 [GPa]
Shear modulus (*G*_12_)	5.52 [GPa]	Density (*ρ*)	7,750 [kg/m^3^]
Poisson ratio (*ν*_12_)	0.31	Poisson ratio (*ν*_21_)	0.16
Piezoelectric Constant (*d*_11_)	400 [pC/N]	Piezoelectric Constant (*d*_12_)	−170 [pC/N]
Permittivity (*ε*_11_ / *ε*_0_)	830 [C/m^2^]	Permittivity (*ε*_22_ / *ε*_0_)	916 [C/m^2^]
**Water (H_2_O)**			
Density (*ρ*)	1,000 [kg/m^3^]	Speed of Sound	1,500 [m/s^2^]

**Table 2.** Comparison of natural frequencies of the cylindrical shell structure in the air, and underwater.

**Mode**	**In the Air (Hz)**	**Underwater (Hz)**	**Reduction rate (%)**
1st	587.4	193.7	67
2nd	617.5	215.9	65
3rd	830.7	351.7	58
4th	1,109.8	617.9	44

**Table 3.** Natural frequencies of the underwater cylindrical shell obtained by FEA and experiment.

**Mode**	**FEA (Hz)**	**Experiment (Hz)**
1st	193.7	208
2nd	215.9	251
3rd	351.7	381
4th	617.9	577
